# Supplementary figures and images for: Effect of electrode size and distance to tissue on unipolar and bipolar voltage electrograms and their implications for a near-field cutoff
Source: Sci Rep. 2024 Nov 8;14:27184. doi: 10.1038/s41598-024-78627-5 (PMC11549492; doi:10.1038/s41598-024-78627-5)

Supplemental Figure 1


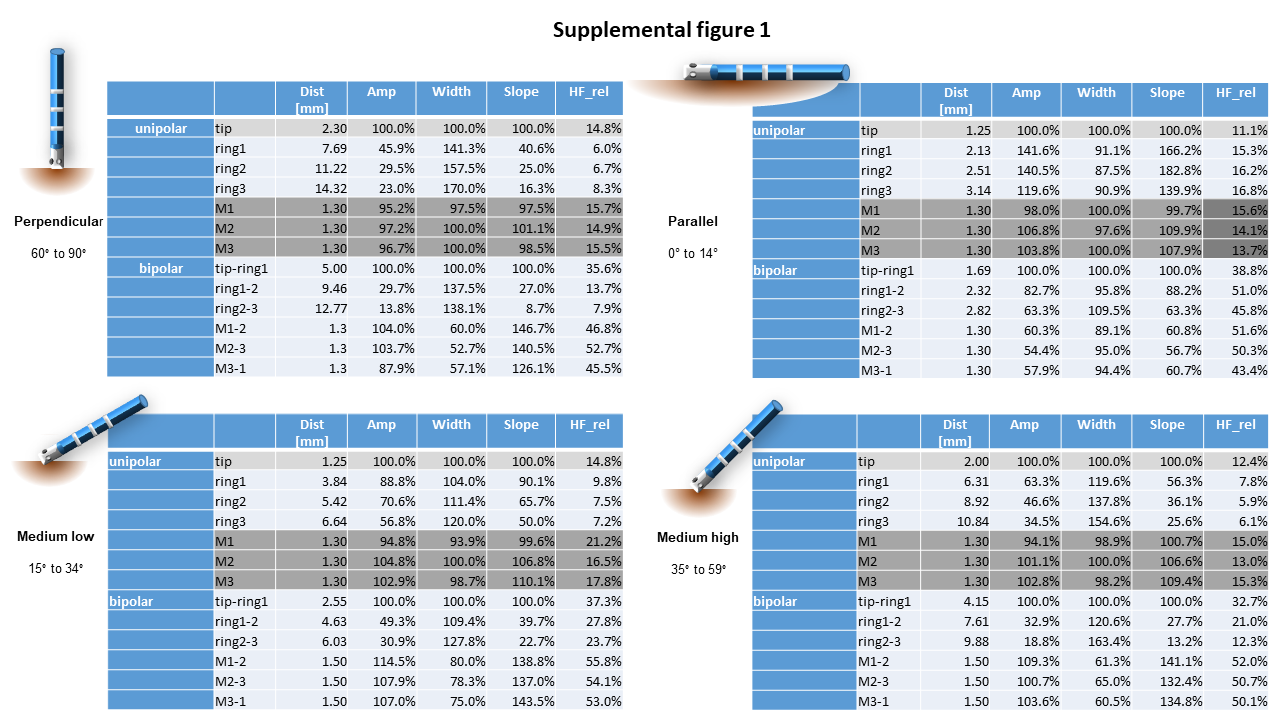


Unipolar and bipolar values for the four grouped catheter orientations.

Supplement: Supplementary file 1 — Supplementary Material 1 [file 41598_2024_78627_MOESM1_ESM.docx]
